# Current Trends in Anaesthesia Monitoring: A Survey Study

**DOI:** 10.4274/TJAR.2025.251987

**Published:** 2026-02-09

**Authors:** Muhammet Selman Söğüt, Yasemin Sincer, Ergün Mendeş, Yavuz Gürkan

**Affiliations:** 1 Koç University Faculty of Medicine Koç University Hospital, Department of Anaesthesiology and Reanimation, İstanbul, Türkiye

**Keywords:** Anaesthesia depth, monitoring in anaesthesia, neuromuscular blockade, nociception monitoring, perioperative care

## Abstract

**Objective:**

This study aims to evaluate the use of anaesthesia depth, nociception, and neuromuscular blockade monitoring among Turkish anaesthesiologists, exploring the frequency of their use, the devices employed, and the barriers to their routine adoption in clinical practice.

**Methods:**

A cross-sectional survey was conducted among 62 anaesthesiologists attending a symposium in İstanbul, Türkiye. Participants were asked about their monitoring practices, devices used, and reasons for not consistently using these technologies. Data were analysed using descriptive statistics and subgroup comparisons based on professional title and hospital type.

**Results:**

Anaesthesia depth monitoring was frequently used by only 37.1% of participants, with cost and availability as major barriers. Nociception monitoring was more commonly used (72.1% frequently) but still faced challenges such as cost and device unavailability. Neuromuscular blockade monitoring was the least used; with 24.2% of respondents never using it. There were no significant differences in responses based on professional title or hospital type.

**Conclusion:**

The study highlights significant variability in the use of advanced monitoring technologies. Barriers such as cost, device unavailability, and reliance on alternative methods hinder their widespread adoption. Addressing these barriers could enhance patient safety and improve perioperative outcomes through more consistent use of monitoring tools.

**Main Points**

**• Inconsistent use of Anaesthesia Depth Monitoring: **Only 37.1% of anaesthesiologists frequently use anaesthesia depth monitoring, with cost and availability being significant barriers. This inconsistency may increase the risk of intraoperative awareness or excessive anaesthetic use.

**• Wider Adoption of Nociception Monitoring:** Nociception monitoring was frequently used by 72.1% of participants, although barriers such as cost and device availability limit its widespread implementation.

**• Low Utilization of Neuromuscular Blockade Monitoring: **Despite its importance in preventing postoperative complications, only 9.7% of anaesthesiologists frequently monitor neuromuscular blockade, with many relying on clinical signs or alternative methods.

**• Barriers to Routine Monitoring:** Cost, device availability, and reliance on traditional methods are the main factors hindering the routine use of advanced monitoring technologies in anaesthesia practice. Addressing these barriers could improve perioperative patient outcomes.

## Introduction

The use of physiological monitoring in anaesthesia has significantly evolved, providing anaesthesiologists with valuable tools to enhance patient safety, optimize anaesthetic dosing, and improve perioperative outcomes. Among these, monitoring technologies for anaesthesia depth, nociception, and neuromuscular blockade have been developed to offer objective assessments that guide intraoperative management. However, despite their potential benefits, adoption and utilization remain inconsistent across different clinical settings.

Anaesthesia depth monitoring aims to reduce the risk of intraoperative awareness, prevent excessive anaesthetic administration, and improve postoperative recovery.^1^ Devices such as Bispectral Index^®^ (BIS), SedLine^®^, Entropy^®^, and NeuroSense^®^ provide quantitative assessments of anaesthetic depth, but their routine use is inconsistent due to concerns regarding cost, accuracy, and clinical necessity.^2, 3^

Nociception monitoring has emerged as a promising tool for individualized analgesia, potentially reducing opioid overuse and postoperative pain. Monitors such as Nociception Level Index^®^ (NOL), Analgesia Nociception Index^®^ (ANI), Surgical Pleth Index^®^ (SPI), and the Response Entropy^®^ monitor aim to provide real-time assessments of intraoperative nociceptive responses.^4, 5, 6, 7^ Their implementation remains limited due to device availability and unclear clinical impact.^2, 3^

Neuromuscular blockade monitoring is recommended to ensure complete recovery from neuromuscular blocking agents, reducing the risk of postoperative residual paralysis, and respiratory complications.^8^ However, studies indicate that many anaesthesiologists continue to rely on clinical signs rather than objective quantitative monitoring, potentially increasing the likelihood of incomplete neuromuscular recovery.

### Study Rationale

Despite the availability of these monitoring technologies, real-world usage patterns, barriers to adoption, and factors influencing anaesthesiologists’ decisions remain poorly understood. While previous studies have assessed specific monitoring modalities, comparative data on all three—anaesthesia depth, nociception, and neuromuscular blockade—are limited. Understanding anaesthesiologists’ monitoring habits and the obstacles they face can help identify strategies to optimize perioperative monitoring and improve patient outcomes.

### Objective

This study aims to evaluate the frequency and patterns of use of anaesthesia depth, nociception, and neuromuscular blockade monitoring among anaesthesiologists. Furthermore, it seeks to identify barriers to routine use and explore whether monitoring practices differ based on institutional setting or professional experience.

## Methods

### Study Design and Setting

This cross-sectional survey study was conducted among Turkish anaesthesiologists working in various hospital settings. Data were collected in İstanbul, Türkiye, on January 25^th^, 2025, during a symposium on regional anaesthesia. The study was conducted in accordance with the Declaration of Helsinki and approved by the Koç University Ethics Committee (approval no.: 2025.037.IRB3.004, date: 23.01.2025).

### Participants

Participants were eligible if they were actively practicing anaesthesiology. No additional exclusion criteria were applied. Informed consent was obtained from the participants.

### Survey Instrument and Data Collection

The survey included questions regarding demographic characteristics (age, gender, professional title, and years of experience), workplace setting, and monitoring practices for anaesthesia depth, nociception, and neuromuscular blockade. Respondents were asked about the frequency of use, the specific monitoring devices employed, and barriers to regular use. The questionnaire was administered in written format and designed to allow multiple-choice selections where applicable. Responses were collected anonymously to reduce response bias.

### Variables and Outcomes

The primary outcomes were the frequency of monitoring anaesthesia depth, nociception, and neuromuscular blockade; the devices used; and the reasons for not consistently using these monitors. Responses for monitoring practices were categorized as frequently, usually, rarely, or never. Barriers to use were assessed using a multiple-choice format, allowing participants to select all relevant reasons.

### Statistical Analysis

Descriptive statistics were used to summarize participant characteristics, monitoring practices, and barriers to use. Categorical variables were presented as frequencies and percentages. Subgroup analyses were performed to compare responses based on professional title (resident vs. specialist) and hospital type (public teaching hospital, public hospital, university hospital). Statistical comparisons were conducted using chi-square tests or Fisher’s exact tests, as appropriate. Results of the statistical test were corrected using the Benjamini-Hochberg method to control the type-one error rate. All statistical analyses were performed using R version 4.4.1 software. An adjusted *P* value <0.05 was considered statistically significant.

## Results

### Participant Characteristics

A total of 70 anaesthesiologists were invited to participate, and 62 completed the survey. The median age of participants was 33.5 years [interquartile range (IQR): 28.5-40.5; range: 28-60], and the median professional experience was 8 years (IQR: 3-13; range: 1-38). Age data were available for 60 participants and all provided their years of experience.

Among those who reported their gender (n = 51), 47.1% (n = 24) were male, and 52.9% (n = 27) were female. In terms of professional title, 37.1% (n = 23) were anaesthesia residents, while 62.9% (n = 39) were specialists. Most participants worked in public teaching hospitals (77.4%, n = 48), followed by public hospitals (17.7%, n = 11) and university hospitals (4.8%, n = 3).

### Monitoring the Depth of Anaesthesia

**Figure 1:**
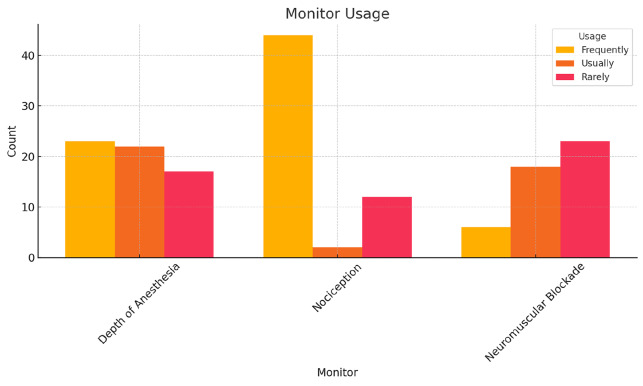
Rates of monitor usage for the depth of anaesthesia, nociception and neuromuscular blockade.

**Figure 2:**
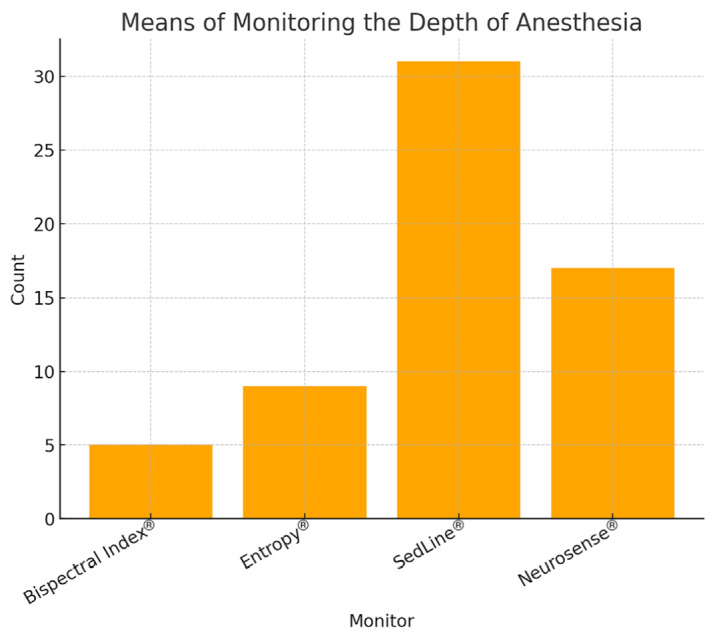
Means of monitoring the depth of anaesthesia.

All participants responded to the questions regarding anaesthesia depth monitoring. Among the respondents, 37.1% (n = 23) reported that they frequently monitor the depth of anaesthesia, 35.5% (n = 22) usually monitor, and 27.4% (n = 17) rarely use depth monitoring (Figure 1). The most used method was SedLine^®^ (50.0%, n = 31), followed by NeuroSense^®^ (27.4%, n = 17), Entropy (14.5%, n = 9), and BIS^®^ (8.1%, n = 5) (Figure 2).

### Monitoring Nociception

**Figure 3:**
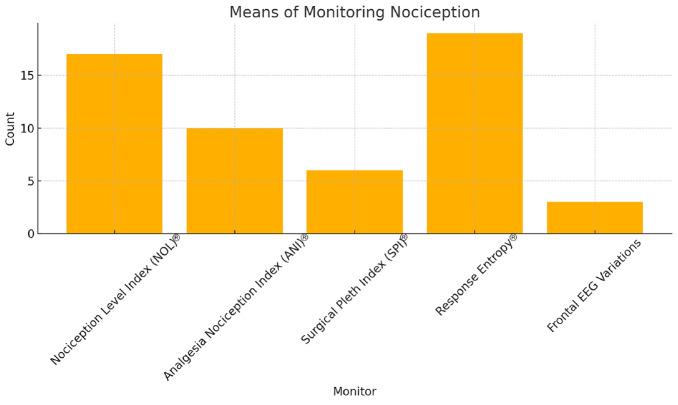
Means of monitoring nociception.

All participants provided responses regarding nociception monitoring. Most participants (72.1%, n = 44) reported frequently using nociception monitoring, while 3.3% (n = 2) usually did, 19.7% (n = 12) rarely used it, and 4.9% (n = 3) never used it (Figure 1). The most reported nociception monitoring methods were response entropy (34.5%, n = 19), NOL^®^ (30.9%, n = 17), ANI^®^ (18.2%, n = 10), SPI^®^ (10.9%, n = 6), and frontal EEG variations (5.5%, n = 3). Seven participants did not report the method they use (Figure 3).

### Monitoring Neuromuscular Blockade

All participants answered the questions on neuromuscular blockade monitoring. Neuromuscular blockade monitoring was frequently performed by 9.7% (n = 6) of respondents, while 29.0% (n = 18) reported usually using it, 37.1% (n = 23) rarely used it; and 24.2% (n = 15) never monitored neuromuscular function (Figure 1).

### Reasons for Not Always Using Anaesthesia Depth Monitoring

**Figure 4:**
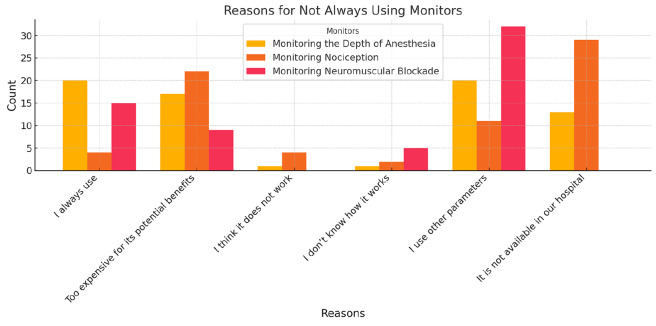
Reasons for not always using monitors for the depth of anaesthesia, nociception, and neuromuscular blockade.

All participants responded to this multiple-choice question. Only 32.3% (n = 20) of respondents reported that they always use anaesthesia depth monitoring, while 67.7% (n = 42) do not. The most frequently cited barriers were cost (27.4%, n = 17) and lack of availability (21.0%, n = 13). Additionally, 32.3% (n = 20) of respondents reported that they use other parameters instead of a depth monitor. Only 1.6% (n = 1) believed anaesthesia depth monitors were ineffective, and another 1.6% were unfamiliar with their function (Figure 4).

### Reasons for Not Always Using Nociception Monitoring

All participants answered this question. A minority of respondents (6.5%, n = 4) reported always using nociception monitoring, while the majority (93.5%, n = 58) did not. The most common reasons for not using nociception monitoring were cost (35.5%, n = 22) and lack of availability (46.8%, n = 29). Additionally, 6.5% (n = 4) expressed doubt about its efficacy, 3.2% (n = 2) stated they did not know how it worked, and 17.7% (n = 11) reported relying on other parameters (Figure 4).

### Reasons for Not Always Using Neuromuscular Blockade Monitoring

All participants answered this question. Among participants, 24.2% (n = 15) reported always using neuromuscular blockade monitoring, while 75.8% (n = 47) did not. The most cited reason was reliance on other parameters (51.6%, n = 32), followed by lack of knowledge (8.1%, n = 5) and cost concerns (14.5%, n = 9). Only 1.6% (n = 1) cited unavailability, and another 1.6% (n = 1) doubted its effectiveness (Figure 4).

### Subgroup Analysis

Subgroup analyses were performed to assess whether responses varied based on the participants’ professional title (resident vs. specialist) and hospital type (public teaching hospital, university hospital, or public hospital). No statistically significant differences were observed between these groups in terms of the frequency of monitoring anaesthesia depth, nociception, and neuromuscular blockade, the methods used for monitoring, or the reasons for not always using these monitors (Figure 1).

## Discussion

This cross-sectional survey provides insights into the monitoring practices of anaesthesiologists regarding anaesthesia depth, nociception, and neuromuscular blockade. The findings demonstrate significant variability in the use of monitoring technologies, with barriers such as cost, availability, and reliance on alternative methods influencing their adoption.

Among the surveyed anaesthesiologists, anaesthesia depth monitoring was not universally practiced, with only 37.1% reporting frequent use. Despite growing evidence supporting its role in reducing intraoperative awareness and optimizing anaesthetic dosing, its use remains inconsistent, possibly due to concerns about reliability, cost, or necessity in routine cases.^1, 2, 3,9,10^ The lack of consistent monitoring may lead to inadequate anaesthetic depth, increasing the risk of intraoperative awareness or excessive anaesthetic administration, which can contribute to delayed recovery and hemodynamic instability.^11, 12^

Nociception monitoring was more widely practiced than anaesthesia depth monitoring, with 72.1% of respondents reporting frequent use. Interestingly, the most commonly used nociception monitor was response entropy, a feature of anaesthesia depth monitors, rather than a dedicated nociception monitor. This suggests that many anaesthesiologists may be relying on anaesthesia depth monitors for nociception assessment, potentially due to familiarity or availability. While numerous studies highlight the potential of nociception monitoring in optimizing opioid administration and improving postoperative pain outcomes. Limited adoption of dedicated nociception monitors could result in suboptimal intraoperative analgesia, leading to either excessive opioid use and its associated side effects or insufficient analgesia, increasing postoperative pain and opioid requirements.^4, 5, 6, 7^

Despite being the oldest monitoring modality, neuromuscular blockade monitoring was the least frequently used modality, with only 9.7% of participants frequently monitoring neuromuscular function and 24.2% never using it.^13^ While some anaesthesiologists may rely on clinical assessments or qualitative nerve stimulators, objective monitoring with quantitative train-of-four or electromyography is strongly recommended to ensure complete recovery from neuromuscular blockade. The lack of routine neuromuscular monitoring increases the risk of residual paralysis, which has been linked to postoperative respiratory complications, including hypoxia, airway obstruction, and an increased need for postoperative ventilatory support.^14, 15^

The most frequently cited barriers to routine monitoring were cost and device unavailability, particularly for nociception and anaesthesia depth monitoring. Cost was a reported limitation for 27.4% of respondents in anaesthesia depth monitoring and 35.5% in nociception monitoring, while device unavailability was a significant concern for 46.8% of respondents regarding nociception monitors. These findings align with previous research highlighting economic constraints as a major factor in the underutilization of advanced monitoring technologies, particularly in resource-limited settings.^2, 3^

A considerable proportion of anaesthesiologists reported relying on alternative parameters rather than dedicated monitors. This suggests that practitioners may favor traditional hemodynamic responses, clinical signs, or subjective assessments over objective monitoring, potentially due to familiarity, skepticism, or concerns about monitor accuracy. While these alternative approaches may provide some clinical guidance, they are less reliable than objective monitoring, increasing the likelihood of imprecise anaesthetic and analgesic management, which may negatively impact patient outcomes.^15, 16^

### Study Limitations

This study has several limitations. First, the survey was conducted among anaesthesiologists attending a regional anaesthesia symposium, which may introduce selection bias, as participants may have a particular interest in advanced monitoring techniques. Second, the study relied on self-reported data, which may be subject to recall bias or social desirability bias. Third, the sample size was relatively small, and findings may not be generalizable to all anaesthesiologists.

## Conclusion

The underutilization of neuromuscular blockade monitoring despite its well-documented benefits in preventing postoperative respiratory complications suggests a need for increased awareness and institutional protocols to promote its routine use. Similarly, expanding access to nociception and anaesthesia depth monitoring could enhance personalized anaesthetic management, but cost considerations must be addressed.

Future studies should explore interventions to improve monitoring adoption, including cost-effectiveness analyses, training programs, and policy-driven implementation strategies. Additionally, investigation into the clinical impact of nociception monitoring on postoperative pain outcomes and the comparative efficacy of different anaesthesia depth monitoring modalities could help define their optimal role in anaesthetic practice.

## Ethics

**Ethics Committee Approval:** The study was conducted in accordance with the Declaration of Helsinki and approved by the Koç University Ethics Committee (approval no.: 2025.037.IRB3.004, date: 23.01.2025).

**Informed Consent:** Informed consent was obtained from the participants.
